# Sequential Model Based Intrusion Detection System for IoT Servers Using Deep Learning Methods

**DOI:** 10.3390/s21041113

**Published:** 2021-02-05

**Authors:** Ming Zhong, Yajin Zhou, Gang Chen

**Affiliations:** Computer Science and Technology College, Zhejiang University, Hangzhou 310027, China; 11521014@zju.edu.cn (M.Z.); yajin_zhou@zju.edu.cn (Y.Z.)

**Keywords:** IoT, Intrusion Detection System, system security, deep learning, sequential model

## Abstract

IoT plays an important role in daily life; commands and data transfer rapidly between the servers and objects to provide services. However, cyber threats have become a critical factor, especially for IoT servers. There should be a vigorous way to protect the network infrastructures from various attacks. IDS (Intrusion Detection System) is the invisible guardian for IoT servers. Many machine learning methods have been applied in IDS. However, there is a need to improve the IDS system for both accuracy and performance. Deep learning is a promising technique that has been used in many areas, including pattern recognition, natural language processing, etc. The deep learning reveals more potential than traditional machine learning methods. In this paper, sequential model is the key point, and new methods are proposed by the features of the model. The model can collect features from the network layer via tcpdump packets and application layer via system routines. Text-CNN and GRU methods are chosen because the can treat sequential data as a language model. The advantage compared with the traditional methods is that they can extract more features from the data and the experiments show that the deep learning methods have higher F1-score. We conclude that the sequential model-based intrusion detection system using deep learning method can contribute to the security of the IoT servers.

## 1. Introduction

The Internet of Things plays a significant role in the information age, and it is a momentous part of the new information technology. The Internet of Things has two meanings: First, the central section of the Internet of Things is still the Internet; in other words, it is a network that extends and develops on the basis of the Internet. Second, the users of the Internet of Things extend to any item or object. They exchange information and communicate with each other, that is, things are connected. The IoT server is the functional core of the whole IoT business system. The basic functions of terminal sensor data collection and processing and return of processing results are all implemented by the server. In addition, the key tasks of system operation such as user hierarchical authentication, system management and maintenance, and availability monitoring are all completed by the server.

Security is becoming more and more critical in cyber life because of the increasing advances of techniques. The market of IoT is developing rapidly, the number of terminals has increased sharply, and security risks are high. The proportion of security links in the Internet of Things industry chain is low. The Internet of Things business penetrates into multiple industries and affects people’s lives in all directions. The corresponding security issues will also bring serious threats, including life and property safety. The security risks of IoT servers are as follows [1]: servers store large amounts of user data, which will become the focus of attacks on IoT systems; virtualization and container technology helps to promote performance while bringing security issues; system infrastructure and components are vulnerable; the IoT business application interface is exposed to public; the application logic is diverse; and risks are easily introduced.

The security issues of the Internet of Things can learn from the knowledge of traditional network security and make corresponding upgrades to the characteristics of the Internet of Things. An important concept in network security is the attack surface, also known as attack surface or attack level, which refers to the attackable location in the network environment where unauthorized users (attackers) can enter or extract data. This is the data flow as well as the attack surface. Reducing the attack surface will inevitably lead to the deterioration of data liquidity. Relatively, the increase of the data liquidity will inevitably lead to the expansion of the attack surface. Therefore, realizing the security of the Internet of Things is actually to minimize the attack surface under the premise of maximizing the protection of the necessary functions.

In the application of the IoT, security protection cannot be fully achieved only by reducing the attack plane. Since the IoT is a sparse network, the reduction of the attack plane is extremely restricted, and intruders will finally find a path to break a certain node in the network. The so-called security hunters are those who use various security tools and methods to search for and eliminate attackers in the network. Usually, security hunters will assume that the current network has been compromised, and then continuously analyze the various monitoring statuses of the current network to find possible intrusions. In the end, invading intruders will be hunted down. This process is repeated to ensure that the security of the IoT is maintained in a continuous and controllable state, and the complete process of the security hunter hunting intruders is called the intruder kill chain. The intruder kill chain was proposed by a security scientist from Lockheed Martin [2]. He learned from the process of eliminating intruders in the military field and summarized the process of eliminating intruders in the security field. For the intruder, the following processes are required to complete the invasion, also known as the intrusion chain: “Reconnaissance, Armed, Distribution, Exploitation, Installation, Command and Control, Target Actions”.

**Figure 1:**
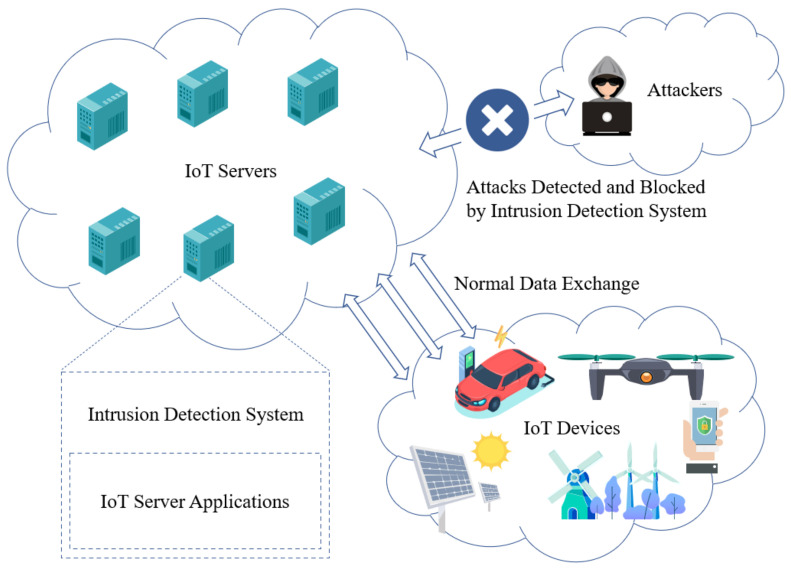
The Scenario of the IDS applied in the IoT network.

In the chain of the security, the first and most important step is how to detect the behavior of the intruder. Only when the intruder is found can the entire killing process be successful. Firewall and Anti-malware software is the first guardian for user terminals. Relatively, IDS (Intrusion Detection System) is the guardian for the Internet servers. Figure 1 shows the scenario which the IDS is applied in the IoT network. In this figure, we can inform that many of the IoT servers and IoT devices are directly exposed to the public Internet because of the feature of remote control. Attackers will capture the vulnerabilities to intrude the IoT servers. An IDS is deeply required to detect and then protect the IoT servers from the attackers.

The use of IDS will not only protect the terminal users but also protect the service providers so that, with the increasing risks on the Internet, the techniques used in IDS should be updated. The techniques of IDS could be classified as anomaly detection and misuse detection. The anomaly detection method is the hotspot because of its capacity to detect unknown risks. In the past, various traditional methods of machine learning had been used in IDS such as SVM, Artificial Neural Networks, Immune Theory, and so on. With years of technique evolution, these methods’ accuracy could reach high levels based on the KDD99 dataset. However, the KDD99 dataset is, in some ways, out of date because it excludes new risks. ADFA-LD dataset takes the place of the old KDD99 dataset, and some traditional methods are not as effective as before.

Deep learning recently takes the place of traditional machine learning methods in many fields. Deep learning uses deep neural networks and various algorithms to link each layer of the network. It is worth noting that these layers usually include an input layer with essential data, which are then analyzed through various hidden layers, and the final stage of the output layer. The model depends on unsupervised functions that form a high-level representation of data from low layers. The newer technologies are being developed in response to the increasing volume of data and the need for more conclusive and accurate assessments.

In this paper, we compare different methods. Moreover, it is essential to measure which method is the best choice for intrusion detection. Finally, we evaluate how the AI contribute to the security of the smart cities. The main works of this paper are described as follows:

- We summarize the risk of the IoT and emphasize the IoT server security.
- We propose two method to improve the efficiency of the intrusion detection system used in IoT server.
- We evaluate the deep learning method and compare them with the traditional method.

The rest of this paper is organized as follows. Section 2 summarizes the related work, emphasizing IoT security and AI methods. Section 3 proposes two deep learning methods for the IDS. Section 4 shows the experiments and results. Finally, Section 5 concludes the paper.

## 2. Related Works

One fundamental and essential component of the information industry is network security since it defends the software and hardware systems of the information industry from the attacks. Nowadays, more and more new data are being generated on the Internet; the demand for protecting data security is growing stronger.

### 2.1. Internet of Things Security

The Internet of Things (IoT) has spawned a new ecosystem of devices connected to the smart city, which is completely different from the architecture that has a centralized core of the system that we are used to in the past [3]. It has become an indispensable part of the smart city. The new world of connected devices will exchange large amounts of sensitive and private data through the embedded device network and other wireless methods [4]. Although the IoT has brought great benefits to individuals, service providers and companies, it also brings a lot of security issues that influence the usability of the system.

Different from ordinary network systems, the IoT is built on the basis of embedded systems, and its communication protocol varies depending on the device and application. Currently, there is no uniform centralized system to construct security measures. Therefore, as the IoT network continues to increase the amount of data exchange, the security risks have reached a new level [5].

In the ordinary network system, malicious code, trojans, hackers, viruses, and other malicious objects are the major elements of the abnormal behaviours and attacks. In the IoT network, it is necessary to emphasize the serious destruction caused by device theft, device manipulation, identity theft, and wiretapping [6]. Once compromised, the IoT may have a serious impact on personal life or the data integrity of the enterprise. For instance, one normal person could be traced by hacker via intruding his/her fitness tracker or a peripheral defense system based on the IoT can be hacked by hackers to illegally access to crucial and confidential areas of the office without authorization. Moreover, IoT vulnerabilities can make it much easier for hackers to achieve the intrusion.

There three major threats of the IoT are as follows [7]:

THEFT
  Steal equipment
  Eavesdrop on data stored in the device
  Theft of intellectual property
FRAUD
  Theft of identity to verify user access
  Fake device credentials to access the server or data repository
CONTROL
  Manipulate data in servers, routers, devices, or clients
  Modify the action of the actuator system
  Force the system to crash to destroy the entire function

Although there are terrible dangers in IoT security, advanced security measures can be used to create a safety net for these devices.

Data encryption

The device can perform identity verification and data encryption before data transmission and exchange. Encryption ensures that even if data were stolen by hackers, they would not be able to access them in their original form. Device identity authentication can ensure that the device is prevented from being manipulated, thereby avoiding the trap of autonomous control by hackers [8].

Sign code certificates

Signing code certificates act as digital signatures, which ensure that only verified code can run on the device, and no one except the editor can destroy or edit the code. This adds another layer of security to IoT devices, which in most cases run on independent platforms [9].

Security on the device side

Cisco estimates that billions of devices and servers would be used and connected to the Internet by 2020, which means that hackers have billions of new attack points. If there is no centralized control to protect these devices, the only way out is to protect the device side [10].

Cloud security

The cloud is the main traffic path for the Internet of Things, so the second perimeter defense that prevents network security must be on the cloud server. Extensive cloud security regulations already exist in the market, which can be adjusted for the IoT environment to achieve the best match [11]. IDS for IoT servers are being studied in recent years, and some deep learning methods such as DBN have been put in use [12].

### 2.2. Traffic Identification

Traffic identification in the network is a critical component of cybersecurity because it triggers red flags to intrude into the network. It is worth noting that the system relies on traditional detection methods, which have become increasingly ineffective owing to the corresponding increase in traffic packets. Traditional methods include port detection. For example, basic HTTP cannot perform as expected because the system follows fewer protocols. Another system applies signature pattern methods that rely on given payload. Importantly, this method can be used in a variety of applications [13].

Researchers have proposed many machine learning algorithms for traffic recognition. Traditional machine learning methods, for example Naive Bayes [14], Random Forests [15], and Decision Tree [16], have been extensively applied in classifying traffic identification. Jun et al. [17] mixed RBM with SVM to detect and identify the network traffic.

Nevertheless, machine learning techniques have brought about advanced methods for analyzing security issues. Deep learning methods can be applied in the field of network security in many ways as well. A recent survey concludes that hybrid methods have been used in intrusion detection system. Different deep learning methods are used in different modules of the whole procedure [18]. The method has proven to be useful for detecting various malicious anomalies based on the traffic of network because it uses some statistical methods to handle the data calculation on the network by evaluating the interrelationships between the neutral points of the system [17]. To identify the anomalies in cybersecurity, it is essential to evaluate multiple variables in cybersecurity.

### 2.3. Factors in Intrusion Detection

First, when the IDS tries to detect an intrusion which aims at the system, there is always the challenge of distinguishing standard data from system abnormal data. To this end, the detection method should have the annotation of the malicious characteristics of data in the intrusion detection system. In addition, the classification system should be designed by appropriate technology that can accurately distinguish two sets of information about normal data and abnormal data. The system uses so-called dimensionality reduction technology, which can automatically calculate the distance between nodes by specific code in the network [19]. In fact, the technology is applied based on the following fundamental assumption: the data integrity and normality are estimated by the consistency of the specific distance between each pair of nodes [20]. In this way, when nodes have a long distance between them, it indicates that the information is abnormal, thus serving as an indication of the existence of malicious and abnormal data. That there are two measurement systems used for this Manhattan distance, which calculates the accumulated distance of the size in the network, and Euclidean distance, which mainly calculates the vector’s size to be evaluated.

Second, deep learning faces the challenge of the integrity of samples used to detect anomalies, which is the so-called ordinary poisoning. The functions used to extract the features are essential because the output is determined by them, especially in unsupervised deep learning. Therefore, this method should guarantee that typical data are not affected; this ensures that abnormal data cannot be hidden in the network’s standard information, thereby making the whole process self-defense. It is worth noting that different methods, including increased traffic and information manipulation, can be used to manipulate the network [21].

### 2.4. Traditional Detection Methods

Most importantly, a variety of methods can use machine learning to detect malicious anomalies in network. The dimensionality reduction of automatic encoder is a method which depends on the components of encoders and decoders [22]. The components also include input layer, output layer, and hidden layers. In addition, the autoencoder uses the following procedures: pre-training, deployment, and fine-tuning.

Of equal importance, Deep Belief Network (DBN) [23] uses the unsupervised RBM layer and the supervised backpropagation network layer and is treated as a traditional deep learning method. There are two types of DBN methods: RBM processes them in an unsupervised way and the following procedure involves the backpropagation algorithm, which involves using the final output of the RBM as the BP layer’s new input and then classifying it using the supervised method.

Therefore, the combination of these two technologies constitutes a mixed-type anomaly detection system. This method depends on reducing the number of dimensions by using an autoencoder to separate vectors [24]. Therefore, the DBN system is used to classify these data through deep learning. Finally, in addition to reducing the time-related complexity in the hybrid system, the accuracy of the detection is also improved.

Protocol identification is a significant threat for traditional detection methods because the research has determined that more than a one eighth of traffic on a network is unknown [25]. However, deep learning methods can perform probability operations on unknown streams, thereby improving accuracy.

### 2.5. Sequential Detection Method

In NLP tasks, the language model represents the probability distribution of the word sequence. The language model based on RNN is not only applied in the NLP tasks but also suitable for other sequential data. Most data in the cyberspace security area are sequential, such as system routine sequence, network request load, and the underlying program code. Due to the long-term dependence problem in sequence modeling of basic RNN models, the vanishing gradient and exploding gradient will cause the instability phenomenon. The LSTM model is chosen in some the relevant papers and experiments [26].

The system routine sequence represents the interaction of the program and system kernel so that it represents the most useful and accurate data in anomaly-based intrusion detection. In the aspect of data acquisition, the real-time traces of the system routine can be easily collected [27]. In addition, the system routine sequence equals the language between the program and system. Under this view, the system routine and its sequence correspond to the word and sentence in natural language, respectively. Based on this, the abnormal system routine sequence is detected.

This sequential model consists of two parts: the front is for language modeling of system routine sequence and the back is for anomaly prediction based on an ensemble of thresholding classifiers. The language model estimates the probability distribution of the system routine in sequence. The data are fed into the model with a one-hot encoding form [28]. In the training part, standard samples are given to the model via the BPTT algorithm [29]. kNN and k-means [30] are chosen to be the classifier at the back-end.

The detection of a web shell needs more steps than backdoors. First, PHP files should be converted into opcode using VLD. Then, the BOW model can be used to make the opcode into a sequence. At last, the sequence can be fed into the language model.

## 3. Deep Learning Methods for IDS Implementation

Developments in information technology have required newer and better methods to analyze how these information systems work. There are various machine learning methods that study the principles of the device. Deep learning is treated as advanced technology and widely deployed in multiple fields, including pattern recognition, natural language processing, and network security. Due to the corresponding increase in the production of data, the ordinary machine learning methods deployed in the field of cybersecurity are gradually unable to work for intrusion detection in the network systems [25]. Therefore, the use of deep learning methods for big data analysis is an innovation that attempts to study the patterns of network connections to detect unauthorized access to computer networks.

Primarily, the system operates according to known principles, including two machine learning methods: probabilistic and deterministic. The deterministic method of machine learning employs small sample datasets and analyzes these datasets to find any deviation from the regular patterns. IT experts will then evaluate this information and develop models for weighted data investigation. Usually, the information in the model is compared with the baseline. Therefore, any abnormal data beyond the average level are deemed to be invasive.

The probabilistic method of machine learning takes another big progress because it can evaluate the patterns involved in the evaluation, and these patterns may not be able to found in deterministic analysis. In fact, the whole system depends on the cluster detecting any strange characters related to the data. The system depends on the unsupervised operation, where the whole system operates independently, and generating the map and ultimately analyzing any abnormal behavior by the same machine, so this method is more effective because the evaluation is conclusive. The exact problem can be determined, by conservative estimates, 90% of the time.

Deep learning has also recently gained attention because of its advantages. This method is dynamical because the system is predictable and can adapt to generated data. It is worth noting that this method uses the output of the top-down method as the input of the bottom-up method. In addition, the model uses linear models to extract features, and these linear models are used as basic functions of layers. These layers depend on each other to form a more in-depth system architecture.

The target of the detection is sequence data consisting of several system-calls so that they can be treated as time-series data. The natural language processing method is a solver for this kind of data. RNN is sufficient to model sequences, but it has some gradient problems, thus we choose an extension named GRU to address these problems. In addition, some variations of CNN would have effectiveness in solving character-level problems, and they can be transformed to adapt to sequence modeling. Thus, we use the Text-CNN model.

### 3.1. Method of Classification

The deep learning scheme of time series classification is roughly performed as follows: the input is a time series, end-to-end training is carried out through a certain neural network algorithm, and the corresponding classification probability is finally output.

The deep learning algorithm for time series classification is divided into two methods: generative and discriminative. The generative formula includes algorithms such as autoencoder and echo state networks, and the discriminant formula includes time series feature engineering and various supervised algorithms, as well as end-to-end deep learning methods. The end-to-end deep learning methods include common algorithms such as feedforward neural networks, convolutional neural networks, and hybrid models.

The evaluation index of the model uses mean per-class error, which refers to the average error rate of each class (Class) in a multi-class scenario.

(1) $$D={\left\{d_{k}\right\}}_{1\leq k\leq K}$$

whew *D* represents for the dataset and $d_{k}$ represents for the elements in the dataset.

(2) $$C={\left\{c_{k}\right\}}_{1\leq k\leq K}$$

where *C* represents for the set of the classes and $c_{k}$ represents for each class type.

(3) $$MPCE=\sum_{1\leq k\leq K}e_{k}/K$$

where *K* represents for each dataset and MPCE represents the expected error rate of a single class in all datasets.

### 3.2. Implementation of the GRU

**Figure 2:**
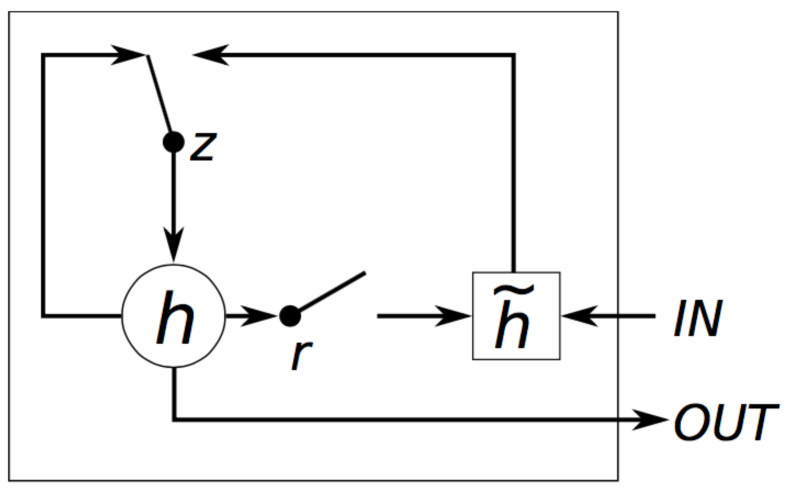
The structure of GRU.

GRU [31] (Gated Recurrent Unit) was proposed by Cho et al. to make each recurrent unit to adaptively capture the dependencies of different time scales. Figure 2 shows the structure of GRU. The figure shows GRU has two gates, a reset gate *r* and an update gate *z*. Intuitively, the reset gate determines how to combine the new input information with the previous memory, and the update gate defines the amount of the previous memory saved to the current time step.

We use the previous state $h_{\left(t-1\right)}$ and the present input $x_{t}$ to obtain the state of the reset gate $r_{t}$ and update gate $z_{t}$.

(4) $$r_{t}=\sigma \left(W_{r}\cdot \left[h_{t-1},x_{t}\right]\right)$$

(5) $$z_{t}=\sigma \left(W_{z}\cdot \left[h_{t-1},x_{t}\right]\right)$$

The reset gate should reset the previous state $h_{t}$ to ${\tilde{h}}_{t-1}$ and concatenate the ${\tilde{h}}_{t-1}$ with $x_{t}$. Then, a tanh function is used to zoom the data to the range of [−1,1].

(6) $${\tilde{h}}_{t}=tanh\left(W_{\tilde{h}}\cdot \left[r_{t}*h_{t-1},x_{t}\right]\right)$$

The ${\tilde{h}}_{t}$ includes the present input data $x_{t}$ and it is pertinent to the present hidden state as it memorizes the present state. Then, we use the update gate to update the state. The update can simultaneously forget and select the memory.

(7) $$h_{t}=\left(1-z_{t}\right)*h_{t-1}+z_{t}*{\tilde{h}}_{t}$$

The output layer uses a sigmoid function.

(8) $$y_{t}=sigmoid\left(W_{o}\cdot h_{t}+b_{y}\right)$$

From Equations (4)–(8) in the forward propagation process, it can be seen that the parameters to be learned are $W_{r}$, $W_{z}$, $W_{\tilde{h}}$, and $W_{o}$. $W_{...}$ are weight matrices, which are updated in the procedure of backward pass.

Training GRU is similar to traditional neural network, using back propagation algorithm, but there are some differences. Because the parameters of GRU are commonly used by all time steps, the gradient of each input does not only depend on the calculation of the current step. This is called Backpropagation Through Time (BPTT). The basic and simplified equation of GRU is

(9) $$s_{t}=f\left(Ux_{t}+Ws_{t-1}\right)$$

(10) $${\hat{y}}_{t}=softmax\left(Vs_{t}\right)$$

The cross entropy loss is defined as follows

(11) $$E_{t}\left(y_{t},{\hat{y}}_{t}\right)\;=-y_{t}\log{\hat{y}}_{t}$$

(12) $$E\left(y,\hat{y}\right)\;=\sum_{t}E_{t}\left(y_{t},{\hat{y}}_{t}\right)=-\sum_{t}y_{t}\log{\hat{y}}_{t}$$

where ${\hat{y}}_{t}$ is the right answer and $y_{t}$ is the prediction. Treating the whole sentence as the training example, the total error is the sum of the errors in each time *t*.

Our goal is to calculate the error gradient first, and then use the gradient descent algorithm to learn parameters. Just as we accumulate errors, we also accumulate the gradients at each time point, for example

(13) $$\frac{\partial E}{\partial W}=\sum_{t}\frac{\partial E_{t}}{\partial W}$$

After calculating the partial derivatives for each parameter *W*, the parameter *W* can be updated and iterated in sequence until the loss converges.

**Figure 3:**
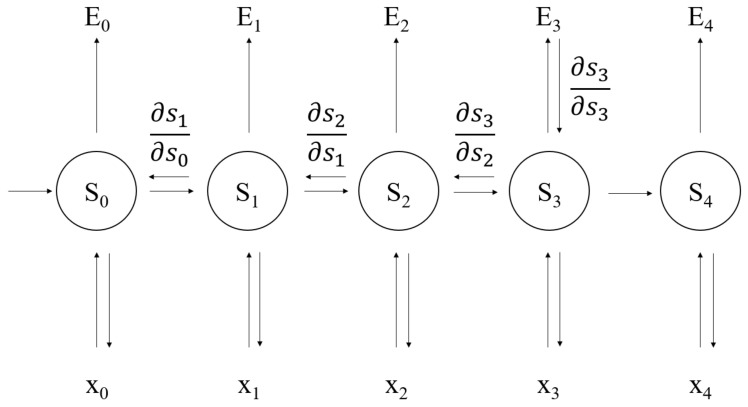
The gradient of error in the BPTT.

The whole procedure of the BPTT is shown in Figure 3. In this figure, $\frac{\partial s_{1}}{\partial s_{0}}$, $\frac{\partial s_{2}}{\partial s_{1}}$, $\frac{\partial s_{3}}{\partial s_{2}}$, $\frac{\partial E_{3}}{\partial s_{3}}$ represent the gradients which pass through time. Each circle $S_{i}$ represents the output value for time *i* and the input value for time i+1 in the hidden layer. $E_{i}$ represents the cross entropy error and $x_{i}$ represents the sample data of the input layer.

### 3.3. Implementation of Text-CNN

**Figure 4:**
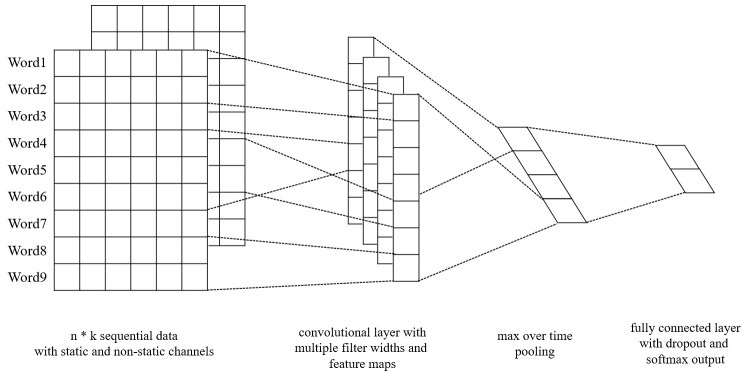
The structure of Text-CNN.

The original CNN (Convolutional Neural Networks) can be recognized as a feature extractor; in other words, it can be used to extract sequence features. On this basis, Yoon proposed Text-CNN [32]. Figure 4 shows the structure of the Text-CNN. Text-CNN uses pre-trained word vectors as embedding layer. For all words represented as a vector, we can get an embedding matrix, and each row in the matrix is a word vector. This matrix can be static or non-static. Then, the procedure of convolution layer outputs a feature map to the pooling layer, and the pooling layer makes the final feature vector.

This model inputs a series of sequence data represented as

(14) $$x_{1:n}=x_{1}⊗x_{2}\;⊗...⊗\;x_{n}$$

Each datum in the sequence is presented as a k-dimension vector; the sequence can be presented as an n*k matrix. A convolution filter w scales n*k operates with the sequence matrix. The f function can be a nonlinear function: tanh, RELU, sigmoid, etc.

(15) $$c_{i}=f\left(w\cdot x_{i:i+h-1}+b\right)$$

A feature map can be produced by this operation

(16) $$c=[c_{1},c_{2},...,c_{n-h+1}]$$

A max-over-time pooling operation is made to obtain the maxc, and it is an essential feature [33]. A softmax, fully connected layer, is used to output the probability distribution of the labels. The fully connected layer uses the dropout strategy for regularization. Element-wise multiplication is used in the output layer, where r represents the masking vector of Bernoulli random variables and z represents the filtered feature map.

(17) y=w·(z∘r)+b

Text-CNN uses the backpropagation algorithm to train the network and the stochastic gradient descent algorithm to update the gradient.

The parameters *W* and *b* of each hidden layer and output layer should be initialized randomly and the forward calculation is used to get the loss $\delta^{i,L}$ of the output layer.

Each layer should perform backpropagation as follows. Fully-connected layer:

(18) $$\delta^{i,l}={\left(W^{l+1}\right)}^{T}\delta^{i,l+1}⊙\sigma^{\prime }\left(z^{i,l}\right)$$

Convolution layer:

(19) $$\delta^{i,l}=\delta^{i,l+1}*rot180\left(W^{l+1}\right)⊙\;\sigma^{\prime }\left(z^{i,l}\right)$$

Pooling layer:

(20) $$\delta^{i,l}=upsample\left(\delta^{i,l+1}\right)⊙\;\sigma^{\prime }\left(z^{i,l}\right)$$

Then, *W* and *b* should be updated as follows.

Fully-connected layer:

(21) $$W^{l}=W^{l}-\alpha \sum_{i=1}^{m}\delta^{i,l}{\left(a^{i,l-1}\right)}^{T},\;b^{l}=b^{l}-\alpha \sum_{i=1}^{m}\delta^{i,l}$$

Convolution layer:

(22) $$W^{l}=W^{l}-\alpha \sum_{i=1}^{m}\delta^{i,l}*a^{i,l-1},\;b^{l}=b^{l}-\alpha \sum_{i=1}^{m}\sum_{u,v}{\left(\delta^{i,l}\right)}_{u,v}$$

For Equations (18)–(22), *m* represents the number of samples, *i* represents iteration counts, *W* represents weights matrix, *b* represents bias vector, δ represents the gradient, α represents iteration step, *l* represents the current layer, σ represents the activation function, and *a* represents the output of the forward pass.

### 3.4. System Definition

**Figure 5:**
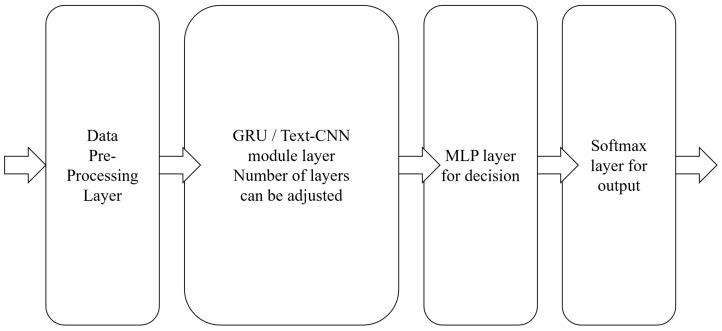
The model of the system.

To build a deep neural network for detecting network intrusion, each component can be regarded as a module of the network, and multiple modules are in a cascade relationship. By combining these modules, the overall structure of the intrusion detection system we propose is shown in Figure 5. The system is composed of a preprocessing module, a feature extraction module, a classification decision module, and an output module. The preprocessing module processes the data into normalized values suitable for input to the neural network, without changing the dimensions of the data. The feature extraction module is composed of one or more layers of GRU (or Text-CNN), which is mainly used to extract and store features and is the core part of the system. The classification decision module is an n-layer perceptron model, which mainly performs nonlinear mapping on the output information of the feature extraction module to realize classification decision. The output module uses softmax regression to normalize the final classification probability.

Among the above modules, the feature extraction module has a greater impact on performance. GRU and Text-CNN have different neural network structures. The former has a memory function, but the structure is also complex and computationally expensive, while in the latter it is easy to stack multiple layers. The deep neural network follows the idea of modular design, and each module can independently process the input data. In actual experiments, the feature extraction module is replaced with other traditional machine learning algorithms. These modifications inevitably affect the experimental results, so that the impact of different algorithms on the detection results can be evaluated. These conditions can be evaluated in detail through experiments.

To build the core feature extraction module layer, the procedure is as follows:

(a) Define a batch size which describes the order of the forward and backward training.
(b) Define an epoch which describes all the examples to be trained.
(c) Define a learning rate which describes update weights of the training procedure.
(d) Define the dropout layer which can perform regularization when it temporarily removes ignored neurons in the forward procedure.
(e) Define the activation function which can transform the input to output where the whole procedure will repeat.

As a deep neural network, it can be trained by stochastic gradient descent with momentum. To improve efficiency, cross entropy is used as the loss function. The algorithm is shown below (Algorithm 1).
**Algorithm 1** Backpropagation weight update algorithm.**Input:** Training dataset $D=\left\{\left(x_{1},y_{1}\right),\left(x_{2},y_{2}\right),\ldots ,\left(x_{n},y_{n}\right)\right\}$   Initial model parameter θ   Learning rate *E*   Momentum α   Batch size *m*
**Output:** Updated model parameter $\theta^{\prime }$ 1: initial velocity *v* = 0 2: **repeat** 3: Randomly select *m* examples $\left\{\left(x_{1},y_{1}\right),\left(x_{2},y_{2}\right),\ldots ,\left(x_{m},y_{m}\right)\right\}$ 4: Compute temporary update $\tilde{\theta }\leftarrow \theta +\alpha v$ 5: Compute gradient $g\leftarrow \frac{1}{m}\nabla_{\tilde{\theta }}\sum_{i}L(f(x_{i};\tilde{\theta }),y_{i})$ 6: Compute velocity update v←αv−Eg 7: Apply the update θ←θ+v 8: **until** Complete the iterations 9: Output the parameter $\theta^{\prime }\leftarrow \theta$


## 4. Results and Discussion

### 4.1. Datasets

The intrusion detection system aims to assure a safe connection in the network with various nodes from potential attackers by classifying the received data packets into normal and abnormal types [25]. This paper compares the accuracy with deep learning and traditional methods on the KDD-99 and ADFA-LD datasets.

#### 4.1.1. KDD99

The KDD99 [34] dataset was released by the US Department of Defense Advanced Research Projects Agency (DARPA). It is an intrusion detection and evaluation project at MIT Lincoln Laboratory, which collected TCP dump network connection and system audit data and simulated various user types and network traffic and attack methods to make it similar to a real network environment. A network connection is defined as a sequence of TCP packets from start to end within a specified period, and, during this time, data are transferred from the source IP address to the destination IP address under a predefined protocol. Each network connection is marked as usual or attack, the anomaly type is subdivided into four categories, with a total of 39 attack types: 22 attack types appear in the training set and 17 unknown attack types appear in the test set.

The three chosen types of attacks in the KDD99 dataset are the following:

**DOS:***Denial of Service* Attack is performed by the attacker who expends all the network or computing resources, resulting in the servers being to. busy to handle normal requests.

**U2R:***User to Root* Attack is an unauthorized access to local superuser privileges by a local unprivileged user.

**R2L:***Remote to Local* Attack is an unauthorized access from a remote machine to a local machine.

The KDD-99 dataset has a problem that the data are imbalances. The synthetic minority oversampling technique [22] should be used to solve this problem and the data summary is listed in Table 1.

Table 2 shows the features used in the evaluation.

#### 4.1.2. ADFA-LD

ADFA-LD [35] host-based intrusion detection dataset is a widely used to test the IDB published by the Australian Defence Force Academy. It instantly records the Linux system-calls from a period. The Linux kernel provides a series of standard interfaces for the communication between the user mode and kernel mode. These interfaces contain a limited reference to hardware devices for the user-mode programs, such as applying the system resources, reading and writing devices, creating new processes, etc. The user-mode programs make requirements and kernel responses. Each system-call has a unique number that can use one-hot encoding on the sequence.

Table 3 shows the attack payloads of the dataset; it includes the most common Linux-based servers function. The brute force of the password is a significant label to show the server is under intrusion. The permission upgrade attack can be detected as a new superuser account being created. Meterpreter and web shell payloads represent the long-term persistent attack. Table 4 shows the data structure of the distributions.

### 4.2. Data Pre-Process of KDD99 Dataset

Since the KDD99 dataset contains symbolic data attributes, it should be pre-processed to the appropriate form. For continuous feature attributes, the measurement methods of each attribute are different. Generally speaking, the smaller is the unit of measurement used, the larger is the possible value range of the variable, and the greater is the impact on the result. To avoid the influence of the difference in attribute measurement, it is necessary to standardize the attribute value.

#### 4.2.1. Quantize the Symbolic Data to Numeric

One-Hot encoding is also known as one-bit effective encoding. The method uses N-bit status registers to encode N states. Each state has its own independent register bit, and, at any time, only one is valid.

#### 4.2.2. Numerical Standardization

First, calculate the average value and average absolute error of each attribute.

(23) $${\bar{x}}_{k}=\frac{1}{n}\sum_{i=1}^{n}x_{ik}$$

(24) $$S_{A}=\sqrt{\frac{1}{n}\sum_{i=1}^{n}{\left(x_{ik}-{\bar{x}}_{k}\right)}^{2}}$$

where $x_{k}$ stands for the average value of the attribute *k*, $S_{k}$ stands for the average absolute error of the attribute *k*, and $x_{ik}$ stands for the attribute *k* of the record *i*. Then, standardize each data record.

(25) $$Z_{it}=\frac{x_{it}-{\bar{x}}_{i}}{S_{t}}$$

where $Z_{ik}$ stands for the attribute *k* of the record *i* after standardization.

#### 4.2.3. Numerical Normalization

Normalize each value after standardization into the range [0,1].

(26) $$x^{*}=\frac{x-min}{max-min}$$

where *max* stands for the maximum value of the sample data and *min* stands fo the minimum value.

### 4.3. Data Pre-Process of ADFA-LD Dataset

The ADFA-LD dataset has completed the characterization of various system calls and marked the attack types. The token set composed of syscall api is essentially a word sequence composed of words, and we can perform feature engineering on the samples through word models.

#### 4.3.1. TF-IDF Term Weighting

In the large text corpus, some words appear many times (e.g., “for”, “if”, and “while” in the ADFA-LD dataset), and they carry a small amount of information. We cannot directly use the frequency of these words in the classifier, which would reduce the terms that we are interested in but the frequency is very small. We need to further reweight the count frequency of the feature into a floating point number to facilitate the use of the classifier. This step is completed by TF-IDF conversion [36].

If a word is more common, the denominator is larger, and the inverse document frequency is smaller and closer to 0. The reason for adding 1 to the denominator is to avoid the denominator being 0 (that is, all documents do not contain the word). Here, log means taking the logarithm of the obtained value.

(27) $$tf_{ij}=\frac{n_{i,j}}{\sum_{k}n_{k,j}}$$

(28) $$idf_{i}=\log\frac{\left|D\right|}{\left|\left\{j:t_{i}\in d_{j}\right\}\right|}$$

A high word frequency in a particular document and a low document frequency of the word in the entire document collection can produce a high-weight TF-IDF. Therefore, TF-IDF tends to filter out common words and keep important words. That is, TF-IDF is actually tf∗idf.

#### 4.3.2. Word2Vec

The training process of word2vec is to train a shallow neural network to map each word in the training set to a vector space of a specified dimension [37].

The basic unit of word2vec vectorization is words. Each word is mapped to a vector of a specified dimension, and all words form a word sequence (sentence) to become a vector matrix (the number of words × the specified word2vec embedding dimension). However, the input required by the machine learning algorithm is a one-dimensional tensor. Therefore, we also need to perform feature processing, that is, use the word vector table to perform feature encoding on the original corpus via the method of TF-IDF.

**Figure 6:**
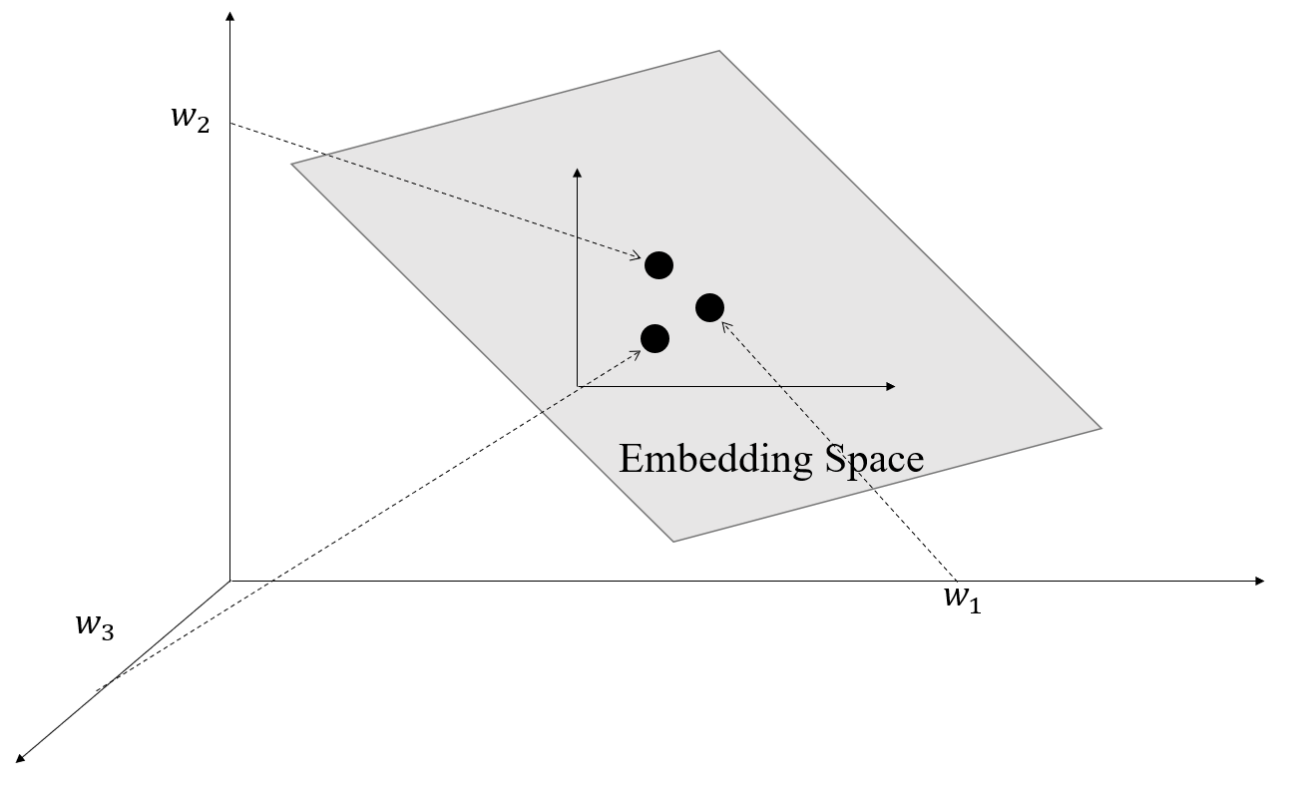
The embedding space of word2vec.

Figure 6 shows the word embedding procedure of word2vec. The procedure first converts each element of the vector from integer to float, which then becomes the representation of the entire real number range. Then, it compresses and embeds the original sparse huge dimension into a smaller dimension space.

### 4.4. Evaluation Indicators

Precision and recall are used in this paper as indicators.

(29) Precision=TP/(TP+FP)

(30) Recall=TP/(TP+FN)

**TP:***True Positive* relates to the num of correctly classified as malicious.

**TN:***True Negative* relates to the num of correctly classified as benign.

**FP:***False Positive* relates to the num of mistakenly classified as malicious.

**FN:***False Negative* relates to the num of mistakenly classified as benign.

(31) $$F1_{score}=2*Precision*Recall/\left(Precision+Recall\right)$$

The F1-score is defined as the weighted harmonic mean of precision and recall [38]. The calculation of F1-score takes into account the accuracy and recall of the test. Accuracy, also known as positive predictive value, is the proportion of positive results that really indicate a positive. Recall rate (also called sensitivity) is the ability to identify a positive result to correctly obtain an accurate positive rate. F1-score reaches the best value when the accuracy and recall are 1. The worst F1-score is obtained when the accuracy and recall are 0.

### 4.5. Hardware and Software Environment

The test environment is shown in Table 5.

### 4.6. Results and Discussion

We chose some traditional machine learning methods, for example C4.5 Decision Tree [39], Naïve Bayes [40], SVM (Support Vector Machine) [41], and the SVM-RBMS [42], to compare with GRU and Text-CNN, which are labeled as new deep learning methods.

**Figure 7:**
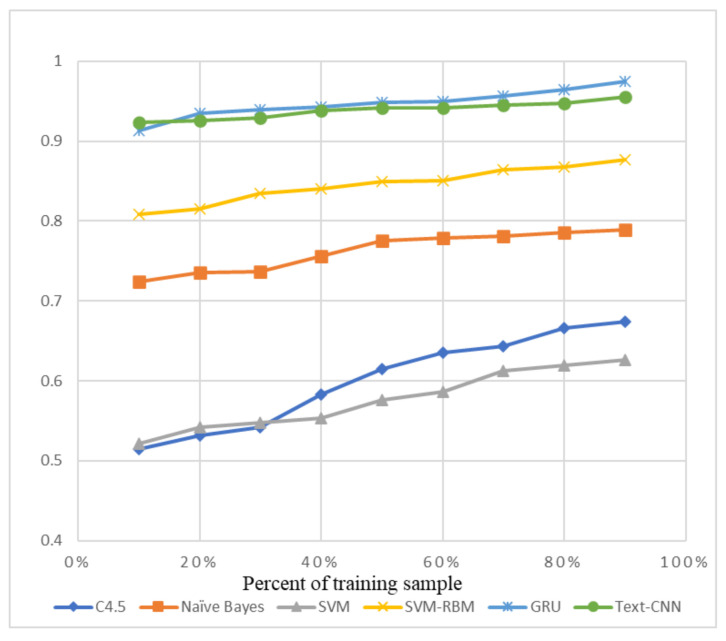
F1-Score of KDD99-Normal.

**Figure 8:**
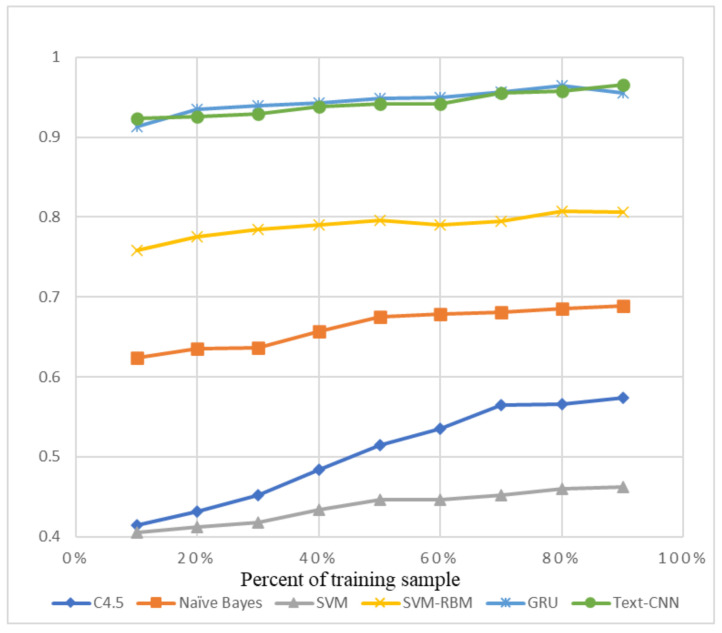
F1-score of KDD99-DOS.

**Figure 9:**
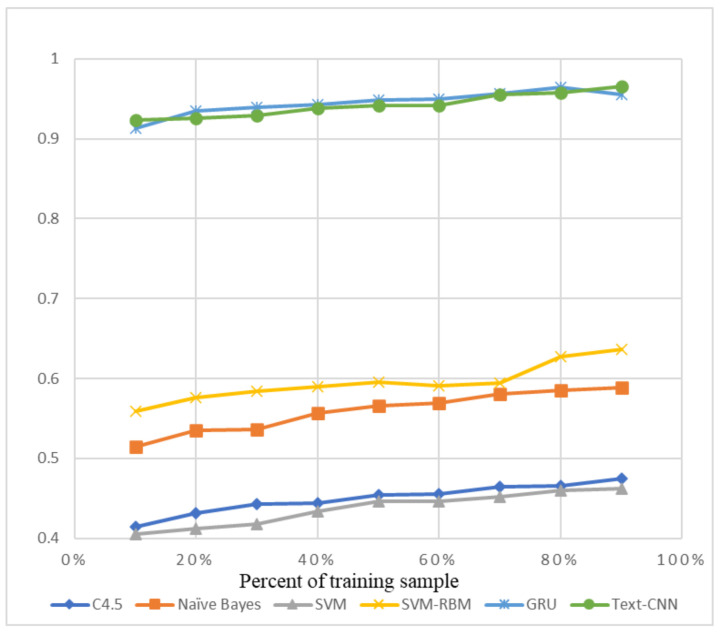
F1-score of KDD99-U2R.

**Figure 10:**
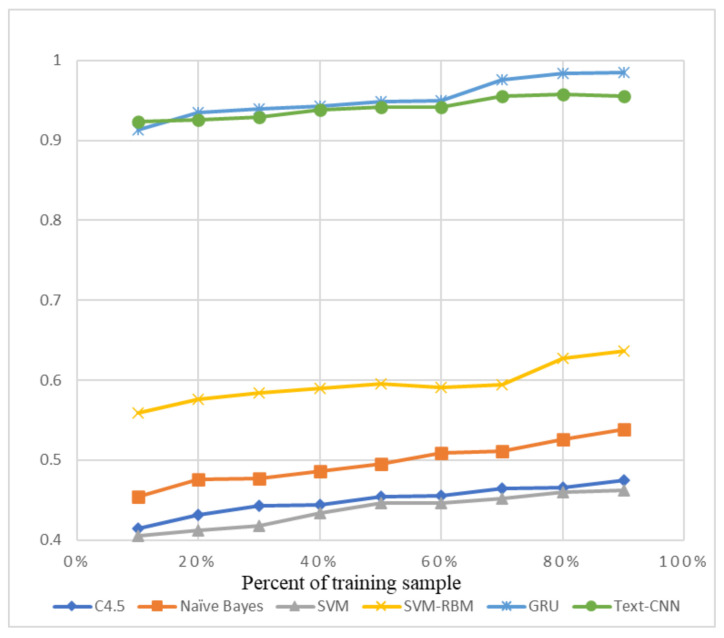
F1-score of KDD99-R2L.

**Figure 11:**
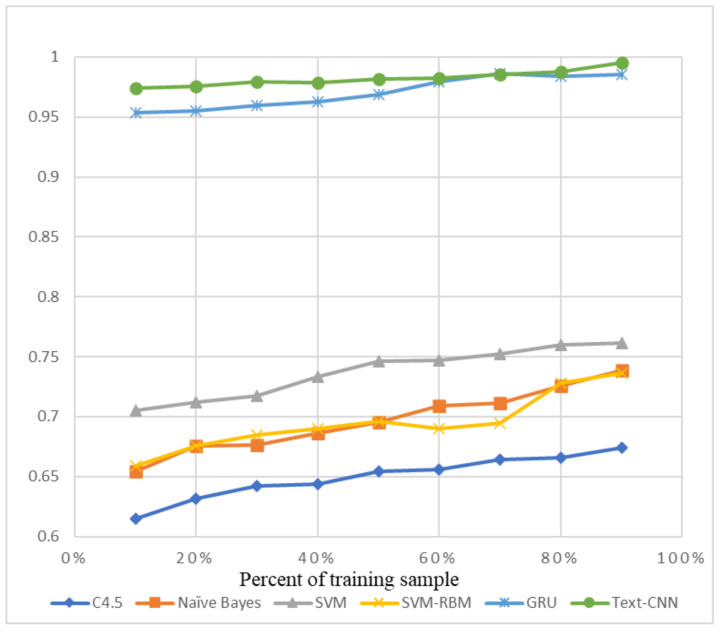
F1-score of ADFA-LD Normal.

**Figure 12:**
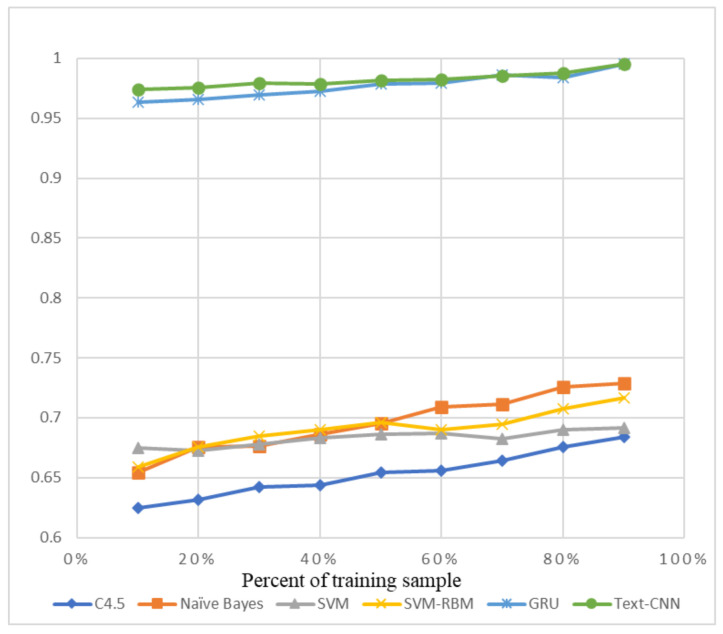
F1-score of ADFA-LD Attack.

The precision and recall were given by the evaluation program, and we used them to calculate the F1-score as the final evaluation standard. The final charts are presented in Figure 7, Figure 8, Figure 9, Figure 10, Figure 11 and Figure 12. Figure 7, Figure 8, Figure 9 and Figure 10 use the KDD99 dataset and separate the attack types. Figure 11 and Figure 12 use the ADFA-LD dataset.

The figures present that new deep learning methods always have the best results. Traditional deep learning method SVM-RBM shows that it is better than the other traditional machine learning methods in some fields. All the other traditional machine learning methods show that they are no longer the right choice for the intrusion detection system.

**Figure 13:**
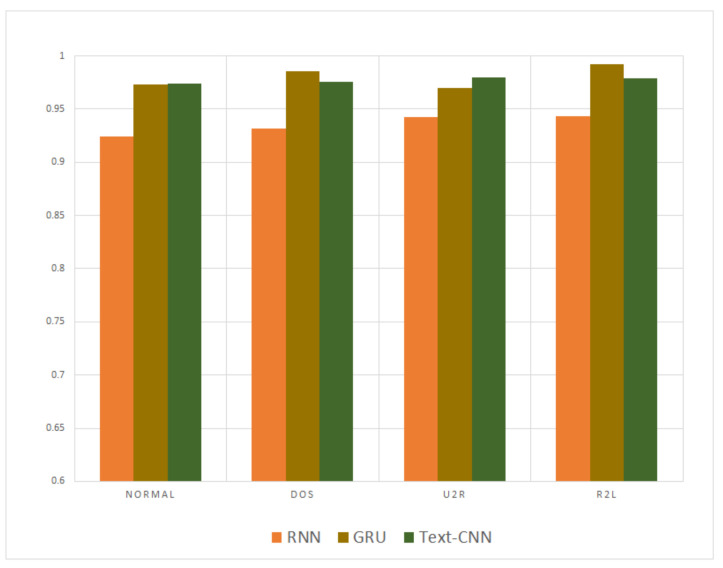
F1-score of the entire KDD99 dataset.

**Figure 14:**
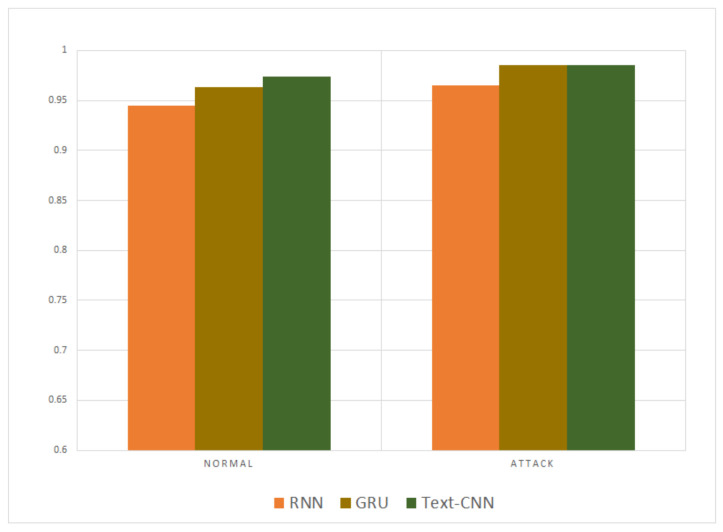
F1-Score of the entire ADFA-LD dataset.

After being compared with the traditional methods, our proposed methods proved their advantages over other former deep learning methods. The GRU is an evolution type of RNN, so RNN method was compared separately to our proposed methods. Figure 13 and Figure 14 compare the KDD99 and ADFA-LD datasets, respectively. The results show that the disparity is not remarkable but still sufficient to show our model is better.

The deep learning methods proposed above have advantages over traditional technologies. First, they can provide accurate signal about malicious behavior, because they can point out the major problem, as well as the root cause of the invasion. Besides, the system depends on machine learning models to detect anomalies; otherwise, it will be difficult for human analysts to notice these problems. In addition, this method can evaluate large amounts of information, which is very inefficient in traditional machine learning methods. The newer technologies enable institutions to formulate better cybersecurity strategies because new deep learning methods are far more efficient than traditional methods. The major benefit of new deep learning methods is that they can fit the variational contexts related to data, thus ensuring that the technology can perform detailed data analysis.

## 5. Conclusions

The construction of the Internet+ era is highly integrated with many new forms of information technology such as IoT, cloud computing, and big data. These are the basis for the realization of intelligent city functions and a complex large-scale system project. There are security risks and vulnerabilities from the sensing and perception layer, communication transmission layer, application layer, intelligent analysis and processing, etc., and they have information security risks that are different from the characteristics of the traditional network era. Once the network security protection cannot be effectively guaranteed, it may cause confusion in management functions, leakage of private information, error in emergency decision-making, frequent occurrence of various accidents, and even local social unrest. Therefore, preventing information security risks is an extremely important. Extraordinarily, the IoT server security should be put in first place. Intrusion detection system is the guardian of the IoT server.

Due to the effectiveness of deep learning in assessing network security, a new use has received much attention. Importantly, the system has achieved a conclusive and detailed assessment of network security. It is worth noting that, due to the increase in data processing, traditional machine learning methods for network security are prominently unable to work efficiently. Nevertheless, the deep learning methods have completely changed the assessment of cybersecurity threats. The system uses multiple methods to identify anomalies in the network, including intrusion detection and flow identification. Nevertheless, the system has specific limitations, including the integrity of the data to generate the input and output. Similarly, due to the need for faster and more useful data evaluation, new deep learning methods are becoming increasingly popular.

## Figures and Tables

**Table 1:** Distribution of attacks.

Type	Data Classified Counts
Normal	552,321
DOS	341,286
U2R	775,352
R2L	653,326
PROBING	754,753
Total	3,077,038

**Table 2:** Evaluated features.

No.	Description	Type
1	HTTP Request Code	String
2	HTTP Packet Length	Long
3	Download/Upload	Boolean
4	HTTP Response Code	Long
5	Attachment Size	Double
6	Attachment Type	String
7	Attachment Count	Long
8	Ratio of Packets with the Same Dest IP	Double
9	Ratio of Send-to-receive Data with the Same Subscriber Identity	Double
10	Count of Hyper-links in HTTP with the Same Subscriber Identity	Long
11	Count of Packets in HTTP with the Same Subscriber Identity	Long
12	Count of Received Data with the Same Subscriber Identity	Long
13	Count of Sent Data with the Same Subscriber Identity	Long
14	Ratio of Sent/Received Packets with the Same Subscriber Identity	Double
15	Packets Received with the Same IMSI	Double

**Table 3:** Attack payloads.

Payload	Vector	Sample
Password	Hydra-FTP	162
Password	Hydra-SSH	148
SU and Root	Client-Poisoning	91
Meterpreter Samples of Java	TikiWiki	125
Meterpreter Samples of Linux	Client-Poisoning	75
Webshell Samples	PHP file include	118

**Table 4:** Data structure.

Data Type	Trace Count
Training Data Labelled Normal	552,321
Validation Data Labelled Normal	341,286
Malicious or Intrusion Data	10 Attacks per Table 3

**Table 5:** Hardware and software environment.

No.	Type	Description
1	Operation System	Ubuntu 20.04
2	Experiment Environment	Tensorflow 2.0
3	CPU	Intel 9700 K
4	Core of CPU	8 cores 16 threads
5	RAM	DDR4 32 GB
6	CUDA Card	GTX1070 8 GB
7	Disk	2TB Nvme SSD

## Data Availability

Publicly available datasets were analyzed in this study. This data can be found here: [http://kdd.ics.uci.edu/databases/kddcup99/kddcup99.html] and [https://www.unsw.adfa.edu.au/unsw-canberra-cyber/cybersecurity/ADFA-IDS-Datasets/].

## References

[1] Kumar J.S., Patel D.R. (2014). A survey on internet of things: Security and privacy issues. Int. J. Comput. Appl..

[2] Yadav T., Rao A.M. Technical aspects of cyber kill chain. Proceedings of the International Symposium on Security in Computing and Communication.

[3] Wang W., Xia F., Nie H., Chen Z., Gong Z., Kong X., Wei W. (2020). Vehicle Trajectory Clustering Based on Dynamic Representation Learning of Internet of Vehicles. IEEE Trans. Intell. Transp. Syst..

[4] Wang W., Chen J., Wang J., Chen J., Gong Z. (2019). Geography-aware inductive matrix completion for personalized Point-of-Interest recommendation in smart cities. IEEE Internet Things J..

[5] Wang W., Chen J., Wang J., Chen J., Liu J., Gong Z. (2020). Trust-Enhanced Collaborative Filtering for Personalized Point of Interests Recommendation. IEEE Trans. Ind. Inf..

[6] Chahid Y., Benabdellah M., Azizi A. Internet of things security. Proceedings of the 2017 International Conference on Wireless Technologies, Embedded and Intelligent Systems (WITS).

[7] Alaba F.A., Othman M., Hashem I.A.T., Alotaibi F. (2017). Internet of Things security: A survey. J. Netw. Comput. Appl..

[8] Conti M., Dehghantanha A., Franke K., Watson S. (2018). Internet of Things security and forensics: Challenges and opportunities. Future Gener. Comput. Syst..

[9] Kouicem D.E., Bouabdallah A., Lakhlef H. (2018). Internet of things security: A top-down survey. Comput. Netw..

[10] Bertino E., Islam N. (2017). Botnets and internet of things security. Computer.

[11] Gupta K., Shukla S. Internet of Things: Security challenges for next generation networks. Proceedings of the 2016 International Conference on Innovation and Challenges in Cyber Security (ICICCS-INBUSH).

[12] Thamilarasu G., Chawla S. (2019). Towards deep-learning-driven intrusion detection for the internet of things. Sensors.

[13] Peng H., Liu C., Zhao D., Han J. (2019). Reliability analysis of CPS systems under different edge repairing strategies. Phys. A Stat. Mech. Its Appl..

[14] Kruegel C., Mutz D., Robertson W., Valeur F. Bayesian event classification for intrusion detection. Proceedings of the 19th Annual Computer Security Applications Conference.

[15] Sinclair C., Pierce L., Matzner S. An application of machine learning to network intrusion detection. Proceedings of the 15th Annual Computer Security Applications Conference (ACSAC’99).

[16] Zhang J., Zulkernine M. A hybrid network intrusion detection technique using random forests. Proceedings of the First International Conference on Availability, Reliability and Security (ARES’06).

[17] Yang J., Deng J., Li S., Hao Y. (2017). Improved traffic detection with support vector machine based on restricted Boltzmann machine. Soft Comput..

[18] Aldweesh A., Derhab A., Emam A.Z. (2020). Deep learning approaches for anomaly-based intrusion detection systems: A survey, taxonomy, and open issues. Knowl. Based Syst..

[19] Peng H., Kan Z., Zhao D., Han J. (2019). Security assessment for interdependent heterogeneous cyber physical systems. Mob. Netw. Appl..

[20] Greche L., Jazouli M., Es-Sbai N., Majda A., Zarghili A. Comparison between Euclidean and Manhattan distance measure for facial expressions classification. Proceedings of the 2017 International Conference on Wireless Technologies, Embedded and Intelligent Systems (WITS).

[21] Peng H., Liu C., Zhao D., Ye H., Fang Z., Wang W. (2020). Security Analysis of CPS Systems Under Different Swapping Strategies in IoT Environments. IEEE Access.

[22] Adil S.H., Ali S.S.A., Raza K., Hussaan A.M. (2014). An Improved Intrusion Detection Approach Using Synthetic Minority Over-Sampling Technique and Deep Belief Network.

[23] Hinton G.E. (2009). Deep belief networks. Scholarpedia.

[24] Tolstikhin I., Bousquet O., Gelly S., Schoelkopf B. (2017). Wasserstein auto-encoders. arXiv.

[25] Abubakar A.I., Chiroma H., Muaz S.A., Ila L.B. (2015). A Review of the Advances in Cyber Security Benchmark Datasets for Evaluating Data-Driven Based Intrusion Detection Systems.

[26] Greff K., Srivastava R.K., Koutník J., Steunebrink B.R., Schmidhuber J. (2016). LSTM: A search space odyssey. IEEE Trans. Neural Netw. Learn. Syst..

[27] Bowman I.T., Holt R.C., Brewster N.V. Linux as a case study: Its extracted software architecture. Proceedings of the 1999 International Conference on Software Engineering (IEEE Cat. No. 99CB37002).

[28] Liang J., Chen J., Zhang X., Zhou Y., Lin J. (2019). One-hot encoding and convolutional neural network based anomaly detection. J. Tsinghua Univ. Sci. Technol..

[29] Chen K., Yan Z.J., Huo Q. A context-sensitive-chunk BPTT approach to training deep LSTM/BLSTM recurrent neural networks for offline handwriting recognition. Proceedings of the 2015 13th International Conference on Document Analysis and Recognition (ICDAR).

[30] Jain A.K. (2010). Data clustering: 50 years beyond K-means. Pattern Recognit. Lett..

[31] Chung J., Gulcehre C., Cho K., Bengio Y. Gated feedback recurrent neural networks. Proceedings of the International Conference on Machine Learning.

[32] Kim Y. (2014). Convolutional neural networks for sentence classification. arXiv.

[33] Ansari G.J., Shah J.H., Yasmin M., Sharif M., Fernandes S.L. (2018). A novel machine learning approach for scene text extraction. Future Gener. Comput. Syst..

[34] Olusola A.A., Oladele A.S., Abosede D.O. Analysis of KDD’99 intrusion detection dataset for selection of relevance features. Proceedings of the World Congress on Engineering and Computer Science, WCECS.

[35] Xie M., Hu J. Evaluating host-based anomaly detection systems: A preliminary analysis of adfa-ld. Proceedings of the 2013 6th International Congress on Image and Signal Processing (CISP).

[36] Ramos J. (2003). Using TF-IDF to Determine Word Relevance in Document Queries. Proceedings of the First Instructional Conference on Machine Learning.

[37] Goldberg Y., Levy O. (2014). word2vec Explained: Deriving Mikolov et al.’s negative-sampling word-embedding method. arXiv.

[38] Joshi R. (2016). Accuracy, precision, recall & f1 score: Interpretation of performance measures. Retrieved April.

[39] Quinlan J.R. (2014). C4. 5: Programs for Machine Learning.

[40] John G.H., Langley P. (2013). Estimating continuous distributions in Bayesian classifiers. arXiv.

[41] Chang C.C., Lin C.J. (2011). LIBSVM: A library for support vector machines. ACM Trans. Intell. Syst. Technol. TIST.

[42] Hinton G.E., Osindero S., Teh Y.W. (2006). A fast learning algorithm for deep belief nets. Neural Comput..

